# Impact of the COVID-19 Pandemic on the Pediatric Hospital Visits: Evidence from the State of Florida

**DOI:** 10.3390/pediatric14010010

**Published:** 2022-02-01

**Authors:** Hasan Symum, José Zayas-Castro

**Affiliations:** Department of Industrial and Management Systems Engineering, University of South Florida, Tampa, FL 33612, USA; josezaya@usf.edu

**Keywords:** COVID-19, pediatrics, hospital ED visit, medically underserved areas, inpatient care

## Abstract

Although early evidence reported a substantial decline in pediatric hospital visits during COVID-19, it is unclear whether the decline varied across different counties, particularly in designated Medically Underserved Areas (MUA). The objective of this study is to explore the state-wide impact of COVID-19 on pediatric hospital visit patterns, including the economic burden and MUA communities. We conducted a retrospective observational study of pediatric hospital visits using the Florida State all-payer Emergency Department (ED) and Inpatient dataset during the pandemic (April–September 2020) and the same period in 2019. Pediatric Treat-and-Release ED and inpatient visit rates were compared by patient demographics, socioeconomic, diagnosis, MUA status, and hospital characteristics. Pediatric hospital visits in Florida decreased by 53.7% (62.3% in April–June, 44.2% in July–September) during the pandemic. The Treat-and-Release ED and inpatient visits varied up to 5- and 3-fold, respectively, across counties. However, changes in hospital visits across MUA counties were similar compared with non-MUA counties except for lower Treat-and-Release ED volume in April–May. The disproportional decrease in visits was notable for the underserved population, including Hispanic and African American children; Medicaid coverages; non-children’s hospitals; and diagnosed with respiratory diseases, appendicitis, and sickle-cell. Florida Hospitals experienced a USD 1.37 billion (average USD 8.3 million) decline in charges across the study period in 2020. Disproportionate decrease in hospital visits, particularly in the underserved population, suggest a combined effect of the persistent challenge of care access and changes in healthcare-seeking behavior during the pandemic. These findings suggest that providers and policymakers should emphasize alternative interventions/programs ensuring adequate care during the pandemic, particularly for high-risk children.

## 1. Introduction

Since the outbreak of Coronavirus Disease 2019 (COVID-19) in December 2019, the outbreak continued to spread rapidly and became an unprecedented global healthcare emergency spreading over countries and territories [1]. The United States accounts for the highest overall COVID-19 disease burden globally, with 32.4 million cumulative reported cases and 572 thousand (18% of total) deaths [2,3]. Many US states and city health agencies imposed preventive measures such as closing schools, ‘stay at home’ orders, delaying elective care, and prioritizing urgent care to restrict the propagation of the infection and harness precious resources during the early pandemic months. The collective impact of these restrictions has brought about seismic disruptions in daily life and healthcare-seeking behavior across all age groups. Particularly, one profoundly impacted area during the early pandemic period was emergency department (ED) care-seeking behavior, which serves as a safety net for many underserved patient populations. For many underprivileged Americans, including children with low-income and uninsured families, ED has become the only reliable option for receiving medical treatment, mainly due to a lack of adequate access to care [4].

Early evidence suggested a profound impact on patient ED volumes and hospital admissions with up to 60% reduction by late April 2020 [5]. More recent studies reported a widespread reduction in pediatric ED volumes (45–56%) across children’s hospitals and dedicated pediatric ED with a relative increase in visits with chronic conditions during the pandemic months [6,7,8,9,10,11,12]. However, most of the studies reported a decline using data from pediatric centers rather than general EDs, where the vast majority of children with non-emergent and chronic conditions are cared for annually [13]. Furthermore, only one study explored visit patterns using multiple EDs across several states [11]; however, exploring a span of locations under varying levels of public health restrictions might not reflect the actual changes in pediatric ED care-seeking behavior for the certain state. Current studies also did not explore whether the decline in ED visits differentially impacted different counties under the same state directives or whether resource use differed for underserved communities during the pandemic [8,11]. Furthermore, state-specific data characterizing the clinical and financial impact of the COVID-19 pandemic on pediatric care settings are limited [9,12]. An improved understanding of the overall state and granular level impact of the COVID-19 pandemic, specifically on pediatric care, is crucial for the policymakers/providers to better prepare pediatric care delivery in the current pandemic and post-pandemic periods. Therefore, we aimed to explore the impact of the COVID-19 pandemic on pediatric hospital visits and resource usage patterns variation in Florida State, particularly focusing on disparities among medically underserved communities and historically disadvantaged race/ethnicity. 

## 2. Materials and Methods

### 2.1. Study Design and Settings 

We conducted a retrospective observational study of pediatric hospital visits during April–September 2020, relative to the same periods in 2019, using Florida all-payer ED visit and inpatient discharge data. Florida is the third populous US state with varied rural–urban areas, characterized by the large, diverse children population (58.5% children of color and 31.8% Hispanic race children), higher uninsured and Medicaid coverages, high pediatric care regionalization, and bottom quantile ranking on health indicators [14,15,16]. These characteristics make Florida state hospitals suitable to understand the changes in pediatric care behaviors during a pandemic for diverse, disadvantaged communities and populations [4].

The ED visit and inpatient discharge database is an administrative database maintained and certified by the Florida Agency for Health Care Administration (AHCA). The dataset comprises patient-level information on demographic characteristics, insurance status, and *International Classification of Diseases, 10th Revision, Clinical Modification* (*ICD-10-CM*) diagnosis and procedure codes, patient location (zip code), and hospital charges of all Treat-and-Release ED and inpatient visits from 265 acute care hospitals across 67 Florida counties. Designated medically underserved area (MUA) status was determined using the U.S. Health Resources and Services Administration (HRSA) classification (Supplementary Table S1) [17]. Data on hospital characteristics were obtained through a unique identifier hospital linkage between AHCA data and the American Hospital Association annual survey. AHCA databases are considered limited datasets, as determined by the local Institutional Review (IRB) Board, and IRB approval was therefore not required.

We included all pediatric (0–17 years) hospital visits during the COVID-19 pandemic (April–June 2020, and July–September 2020) and Pre-COVID-19 comparator period (April–June 2019, and July–September 2019) with complete administrative data. We excluded residential addresses outside Florida and COVID-19 related hospital visits from the dataset to mimic the pediatric hospital visit environments before and during COVID-19 among Florida residents. Hospitalizations after child delivery and newborns were also excluded for both COVID-19 and Pre-COVID-19 period datasets. Our study considered the pandemic period from 1 April 2020, when the Florida governor implemented state-wide stay-at-home restrictions. In addition, a new separate ICD-10 code for COVID-19 related hospital encounters became effective from 1 April 2020. 

### 2.2. Variables

The outcome variables were the number of Treat-and-Release ED visits and inpatient hospital admissions before and during COVID-19. Treat-and-Release ED visits were identified as visits to the ED of the hospital system who were discharged without being admitted to the inpatient care. We divided hospital visits into two groups of Treat-and-Release encounters and Inpatient hospital stays. Demographic characteristics for each hospital visit included age (0–1, 1–5, 5–10, 11–14, 14–17 years), gender, and race/ethnicity (non-Hispanic White, non-Hispanic African-American, Hispanic, and others). The other patient characteristics were insurance coverage (Medicaid fee for service, Medicaid managed care, commercial, uninsured, others), day of the week (weekend versus weekday), and discharge planning (routine, post-acute facility, died, against medical advice discharge and home health care) and referral by the physician. Hospital-level covariates are children’s hospital status, location, and bed size. In this study, another critical exposure of interest was hospital visit changes in MUA-designated counties in the State of Florida. MUA status for Florida counties was defined as full MUA (entire designated county), partial MUA (some areas designated), and non-MUA (no designation).

The *ICD-10-CM* primary diagnosis and procedures codes were used to characterize hospital visits by patient disease complexity and by medical urgency. Patient complexity for each visit was divided using previously developed 3-tiered categorical variables of without chronic condition, noncomplex chronic condition, and complex chronic condition [12,18,19]. To classify visits by medical urgency, we used the validated NYU-ED Billing Algorithm developed by the New York University Center for Health and Public Service Research [18,19]. This algorithm assigns probabilities to each ED visit as emergent-not preventable/avoidable, emergent but preventable or avoidable, emergent but primary care treatable, and non-emergent [20]. Based on a patient’s primary diagnosis, each visit was also classified into 18 organ systems or disease categories [21]. We also studied several acute and chronic conditions, including appendicitis; asthma; seizure; sickle cell disease, excluding sickle cell traits; and pneumonia, which are disproportionately prevalent and have higher ED utilization tendency among historically disadvantaged racial/ethnic populations. Hospital visits charges in 2019 were adjusted to the year 2020 US dollar (USD) using Consumer Price Index for Hospital and Related Services. 

### 2.3. Statistical Analysis

All analysis and comparison were conducted separately for two groups, Treat-and-Release ED visits and hospital inpatient care. For each hospital visit group, we stratified patients into two different periods (April–June 2020 and July–August 2020) during the COVID-19 pandemic and compared them with the same periods in 2019. These two separate periods were analyzed to approximate pediatric hospital visits impact due to the differences in the restrictive measures. The April–June period was approximated as the early COVID-19 months with stricter restrictive measures with the stay-home orders, elective surgery cancellation, and in-person school closures. The July–September period was approximated as fewer restrictive measures with phase 2 state reopening and in-person school instruction. Treat-and-Release and Inpatients visits were stratified by demographics, patient and hospital characteristics using frequencies as counts and percentages for categorical variables. When comparing hospital visits from the COVID-19 and pre-COVID-19 periods, categorical and continuous values were compared using a Chi-square and Wilcoxon sign rank test, respectively.

Changes in hospital visit patterns were computed as the relative changes (%) for both Treat-and-Release ED and inpatient visit groups. In order to understand the variation in hospital visit behaviors across Florida States counties and hospitals, we compared resident hospital encounters between Pre-COVID-19 and COVID-19 pandemic periods across hospitals and counties. Comparison of hospital visits changes between MUA, partial, and non-MUA counties was evaluated using Kruskal–Wallis test and post hoc pairwise Dunn’s test. All statistical analyses were performed using R studio, and a two-sided *p*-value less than 0.05 was considered statistically significant.

## 3. Results

Our study included 1,191,633 Treat-and-Release ED encounters and 88,127 inpatient visits in our combined study periods. Among these visits, there were 10,227 COVID-19 related hospital visits, of which 830 were inpatient care visits. Excluding non-residents and COVID-19 associated visits, 835,239 and 386,871 hospital visits occurred between April to September in 2019 and 2020, respectively, which revealed a 53.7% decline in 2020. The changes in ED visits were higher (*p* < 0.01) in April–June than in the July–September period. During the April–June period, the overall hospital visit decline was 62.3%, where higher decline (64%) in Treat-and-Release ED visits and a comparably lower (34.1%) decline in hospital inpatient care visits. There was a rebound in July–September, yet visit volume remained 44.2% below, especially 45.8% for Treat-and-Release visits and 19.5% for inpatient visits.

### 3.1. Regional Variation and Disparities in Underserved Communities 

**Figure 1:**
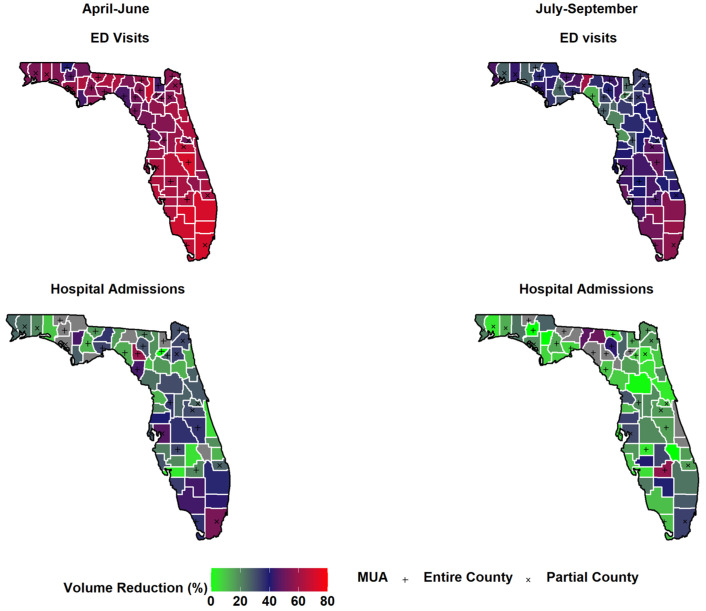
Changes in pediatric hospital visits during COVID-19 pandemic across Florida counties (MUA—Medically underserved Area, ED—Emergency Department).

**Figure 2:**
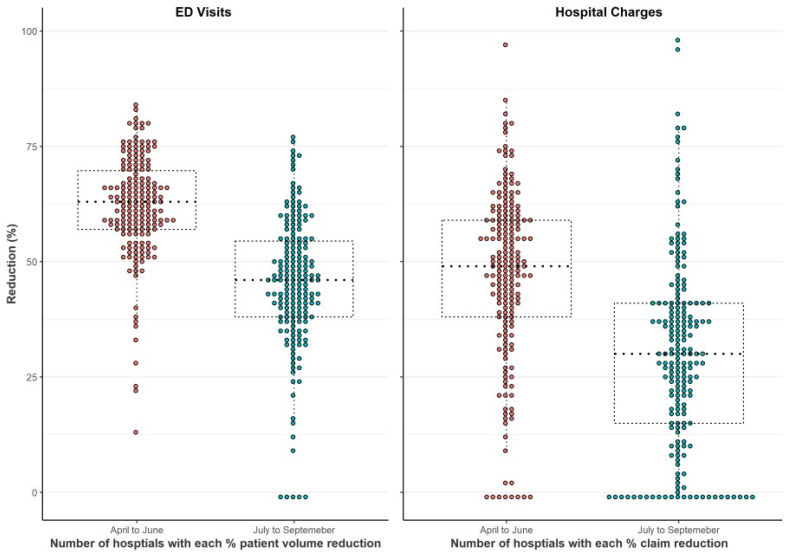
Distribution of pediatric ED visits and hospital claim charge reduction in Florida hospitals. (Dotted line represents Median and Interquartile range).

There was geographical variation in hospital visit change rates across Florida counties during the pandemic period (Figure 1). The decline in Treat-and-Release volume across counties ranged from 40.1% to 72.8% during April–June and ranged from 13.0% to 60.6% in July–September. Changes in pediatric Treat-and-Release hospital visits also varied by MUA designated status. The decline in Treat-and-Release visits volumes in non-MUA counties was significantly higher (Median (Interquartile range), 61.70 (56.48–63.42) vs. 54.6 (51.80–59.10)) than other MUA counties during April–June. However, the decline in Treat-and-Release visits volumes in MUA were similar (*p* = 0.08) to non-MUA counties during July–September. Similarly, however, the difference in inpatient visits changes between MUA and non-MUA counties was not found significant (*p* = 0.32 for April–June, *p* = 0.58 for July–September). Furthermore, the decline in hospital visits varied widely (~6.1–9.6 fold) across hospitals ranged, from low (~8–14%) to high (~77–86%) (Figure 2).

### 3.2. Demographics and Clinical Characteristics Comparison 

Table 1 and Table 2 showed hospital visits in 2019 and 2020 by patient demographics, comorbidity, and visit urgency status. Compared with the previous year, the decline in hospital visits during the COVID-19 pandemic varied across patients’ demographics. The drop in Treat-and-Release hospital visits among children aged 1 to 14 years was (~43.2–68.2%) while hospital visits drop for adolescents (14–17) and infants (0–1) were reported (~33.1–53.4%), respectively. The decline in hospital visits for Hispanic and Non-Hispanic African American race children were (~51.2–69.6%) and (~49.9–66.6%), respectively, while for non-Hispanic White race children were (~38.8–57.5%) during comparison periods. Across insurance coverage categories, Medicaid managed care patients experienced a (~46.9–66.2%) decline in visits, while privately insured patients experienced a (~36.9–56.3%) decline in hospital visits. The declines in hospital visits among children’s hospitals were (~33.3–26.8%), while the non-children’s hospital visits declined by (~44.4–64.3%).

The decline in hospital visits during the COVID-19 pandemic also varied widely by the index disease conditions. The notable decline in Treat-and-Release visits among children with respiratory system disease was 84.1% during April–June and, even in June–September, a 68.0% decline was reported (Supplementary Tables S2 and S3). There was also a sharp decline in hospital visits for children with the central nervous system and infectious diseases. Mental health disorder-related hospital visits declined during the first three months of the pandemic, while there was a slight increase (0.5%) in inpatient visits during July–September. Pediatric ED visits and inpatient care visits with sickle cell conditions were observed an almost steady decline throughout the pandemic periods. Interestingly, although appendicitis-related Treat-and-Release visits were declined throughout the pandemic period, there is an increase in inpatient visits for appendicitis during April–June.

### 3.3. Economic Implications 

The total charges originating from pediatric hospital visits decreased from USD 5.23 billion in 2019 to 3.86 billion in 2020. This represents a 25.2% decrease in total charges during the six months of the pandemic period. The total hospital charges for Florida Medicaid were also decreased by 28.6%, from 3.43 billion in 2019 to 2.45 billion in 2020. Furthermore, for the total 10,227 pediatric COVID-19 diagnoses, the total hospital charges were USD 98.7 million; 71.1 million (average USD 85,625) was for inpatient care, and 27.6 million (average USD 2944) was for Treat-and-Release visits. Out of 166 hospitals operating in both years, 156 (94%) hospitals experienced a reduction in pediatric charges from 2019 to 2020. The average changes in hospital charges for large, medium, and small bed-sized hospitals were USD 20.6 million, 6.6 million, and 2.9 million, respectively. Particularly, Children’s hospitals and public/non-profit hospitals faced the greater financial impact compared with non-children’s hospitals (average 48.5 million vs. USD 8.0 million) and private (average USD 9.6 million vs. USD 7.4 million) hospitals.

## 4. Discussion

Our study has three major results. First, the COVID-19 pandemic led to an unprecedented reduction in pediatric hospital visits in Florida, and this decline varied up to 5-fold across counties. Particularly, MUA counties experienced a similar visits reduction than the non-MUA counties expect comparably lower Treat-and-Release visits during the early pandemic. Second, there was a considerable disproportional decrease in hospital visits for the Non-Hispanic African American and Hispanic children, those who were insured with Medicaid, and conditions related to respiratory, digestive system, appendicitis, and sickle cell disease. Third, Hospitals in Florida faced a substantial financial impact with a total USD 1.37 billion (average USD 8.3 million) pediatric claim reduction in six pandemic months. In general, the study highlights the profound and disproportionate effect of COVID-19 on pediatric hospital care-seeking behavior in Florida State and provides important insights regarding the decline through care access, demographics, and diagnoses.

We observed different patterns of ED visits across the Florida state counties throughout the pandemic, with the steepest decrease in visits across non-MUA counties during the early COVID-19 pandemic. These findings are likely related to the high degree of COVID-19 disease burden and community transmission among rural counties during early pandemic periods. Our findings of a similar decline in ED visits during later pandemic periods in MUA counties as non-MUA counties suggests combined effect a similar elevated COVID-19 disease burden and limited access to pediatric care due to geographical location [22,23]. Furthermore, a similar decline in inpatient hospital visits in MUA counties in both periods suggests persistent rural–urban disparities in pediatric care access and combined with a high degree of pediatric care regionalization, which is likely to be intensified during the COVID-19 pandemic [23,24,25]. These findings highlight the need to develop tailored programs such as community outreach and vigilance, particularly among underserved communities, as the federal and local healthcare systems develop resource strategies for the current and future pandemics.

The higher decline during early pandemic months reported in our study was consistent with prior studies to date [6,7,8,12]. Some extent of the decline during April–June was likely driven by the state lockdown measures, social distancing, especially school closures, as well as the fear and anxiety during the early pandemic periods [8,26,27]. The substantial drop in pediatric respiratory-related hospital visits could be explained by the increased health hygiene (e.g., mask-wearing and handwashing) and limited exposure to environmental infection triggers due to school closures coupled with restricted access to care during the pandemic [26,27,28]. Wearing masks, hand hygiene, and social distancing could contribute not only to the prevention of COVID-19 but also to the decline of other respiratory infectious diseases, including influenza, enterovirus, and all-cause pneumonia [29,30].

However, our findings of the disproportionate decline in pediatric visits for the historically disadvantaged minority-race children and Medicaid suggest a possibility of systematic healthcare inequalities, particularly the persistence of racial-ethnic disparities in pediatric care access [31,32]. The disproportionate decline is also likely fueled by the disproportionate burden of COVID-19 cases within these communities [33,34]. Another concerning finding that emerged from our study was the substantial decline in Treat-and-Release visits for appendicitis while hospital visits increased during April–June. This increase in hospital visits may be caused by the delayed/deferred care in patients during the early months of pandemic and, therefore, increased the inpatient care visits with more complicated conditions [11]. This finding could also be associated with the cases of post-COVID-19 health complications and an increase of the adenovirus associated with acute appendicitis [35,36]. Several case reports have suggested a probable association between COVID-19 and subsequent acute appendicitis [36]. Perhaps the most alarming findings of our study are a substantial steady decline in visits with sickle cell disorder, which is more prevalent in African American children insured with Medicaid and are rarely treated outside of hospital settings [37,38]. These disproportionate declines raise concerns about long-term adverse health outcomes, particularly for the high-risk and medically underserved population. 

Our study offers critical insights into hospital preparedness and suggests opportunities for health officials in preparing emergency care delivery for this ongoing pandemic. The financial impact reported in our study across Florida hospitals just for pediatric visit reduction suggests a major financial crisis for many healthcare systems, particularly in many small rural, public, and children’s hospitals. These findings beg the question of how hospitals can be positioned to financially weather the changes in care delivery patterns during a pandemic. Hospitals could potentially implement strategies to engage certain patients through alternative care settings such as Telehealth and home health care initiatives, particularly for underserved populations [39,40].

This study has several common limitations, most of which are related to a retrospective analysis of administrative claim databases. First, the ACHA database uses ICD codes to classify patients’ information, and the possibility of coding inaccuracy cannot be dismissed. Second, although our study makes a significant contribution of presenting pediatric care information during the COVID-19 pandemic across Florida state hospitals, findings of this study may not be generalizable to other U.S. states or international countries, mainly due to differences in population demographics and pediatric care delivery system. Third, despite a substantial decline in hospital visits reported in our study, hospital visit data do not include whether those visits were the outcome of avoided/deferred care or whether patients visited care in other healthcare facilities (e.g., telemedicine). Finally, the AHCA dataset does not include the exact amount hospitals received from the insurance providers, which may overestimate financial implications for the Hospitals.

## 5. Conclusions

In 2020, pediatric hospital visits declined sharply in Florida State during the first eight months of the COVID-19 pandemic, with notable disproportionate decreases among non-white Hispanic and African American children and children insured with Medicaid compared with other patient populations. In addition, the decline in pediatric hospital visit volumes varied widely across Florida counties. However, the similar decline in hospital visits across Florida MUA counties compared with Florida non-MUA counties except lower Treat-and-Release ED visit volume in April–May suggest a comparable impact on underserved communities, although there were substantially lower COVID-19 cases in these areas. These disproportionate declines in pediatric hospital visit volume across Florida hospitals suggest persistent systematic care disparities for many children, which may be intensified during the pandemic. Therefore, the foregone pediatric hospital visits could be addressed by a tailored intervention program such as community outreach and vigilance, particularly to those who were disproportionately affected. Furthermore, as the federal and local healthcare organizations develop resource strategies for the current and potential pandemics, enhancing primary care capacity and telemedicine could serve as a potential alternative to preventable hospital visits should be prioritized for the most susceptible pediatric patients, particularly among underserved communities. 

## Figures and Tables

**Table 1:** Changes in Treat-and-Release Emergency Department Visits during COVID-19 pandemic by the patient and hospital characteristics.

Variable	April to June				July to September			
	2019 N = 427,714 *n* (%)	2020 N = 155,381 *n* (%)	*p*-Value	Changes (%)	2019 N = 4,391,626 *n* (%)	2020 N = 216,912 *n* (%)	*p*-Value	Changes (%)
Age								
0–1	4097 (1.0)	2436 (1.6)	*p* < 0.01	1661 (40.5)	4807 (1.3)	3084 (1.5)	*p* < 0.01	1723 (35.8)
1–5	150,606 (36.6)	49,543 (33.5)		10,106 (67.1)	135,015 (36.0)	63,810 (31.3)		71,205 (52.7)
5–10	108,403 (26.4)	34,457 (23.3)		73,946 (68.2)68	92,897 (24.7)	47,679 (23.4)		45,218 (48.7)
10–14	70,004 (17.0)	25,090 (16.9)		44,914 (64.2)	64,706 (17.2)	36,768 (18.1)		27,938 (43.2)
14–17	78,247 (19.0)	36,498 (24.7)		41,749 (53.4)	78,048 (20.8)	52,220 (25.7)		25,828 (33.1)
Gender								
Male	199,846 (48.6)	71,153 (49.4)	*p* = 0.34	126,693 (63.4)	181,861 (48.4)	100,689 (49.5)	*p* < 0.01	81,172 (44.6)
Female	211,511 (51.4)	74,871 (50.6)		136,640 (64.6)	193,612 (51.6)	102,872 (50.5)		90,740 (46.9)
Race/Ethnicity								
Non-Hispanic White	142,834 (34.7)	60,742 (41.0)	*p* < 0.01	82,092 (57.5)	133,722 (35.6)	81,898 (40.2)	*p* < 0.001	51,824 (38.8)
Non-Hispanic Black	114,041 (27.7)	38,267 (25.9)		75,774 (66.4)	104,648 (27.9)	52,423 (25.8)		52,225 (49.9)
Hispanic	133,475 (32.4)	40,586 (27.4)		92,889 (69.6)	117,963 (31.4)	57,590 (28.3)		60,373 (51.2)
Others	21,007 (5.1)	8429 (5.7)		12,578 (59.9)	191,410 (5.1)	11,650 (5.7)		7490 (39.1)
Insurance status								
Commercial	79,148 (19.2)	34,557 (23.3)	*p* < 0.01	44,591 (56.3)	72,234 (19.2)	45,577 (22.4)	*p* < 0.01	26,657 (36.9)
Medicaid Fee for service	20,256 (4.9)	7703 (5.2)		12,553 (62.0)	20,737 (5.5)	10,301 (5.1)		10,436 (50.3)
Medicaid managed care	269,952 (65.2)	91,306 (61.7)		178,646 (66.2)	243,411 (64.8)	129,253 (63.5)		114,158 (46.9)
Others	14,245 (3.5)	5716 (3.9)		8529 (59.9)	13,167 (3.5)	8025 (3.9)		5142 (39.1)
Uninsured	27,756 (6.7)	8742 (5.9)		19,014 (68.5)	25,924 (6.9)	10,405 (5.1)		15,519 (59.9)
Physician Referral								
Yes	1062 (0.3)	668 (0.5)	*p* < 0.01	394 (37.1)	1104 (0.3)	987 (0.5)	*p* < 0.01	117 (10.6)
Weekend								
Yes	114,251 (27.8)	43,545 (29.4)	*p* < 0.01	70,706 (61.9)	106,836 (28.5)	56,364 (27.7)	*p* < 0.01	50,472 (47.7)
No	297,106 (72.2)	104,479 (70.6)		192,627 (64.8)	268,637 (71.5)	147,197 (72.3)		121,440 (45.2)
Discharge disposition								
Routine	399,819 (97.2)	141,631 (95.7)	*p* < 0.01	258,188 (64.6)	365,465 M(97.3)	195,627 (96.1)	*p* < 0.01	169,838 (46.5)
Post-acute	2831 (0.7)	2079 (1.4)		752 (26.6)	2474 (0.7)	2356 (1.2)		118 (4.8)
Home Health	68 (0.1)	31 (0.0)		37 (54.4)	55 (0.0)	38 (0.0)		17 (30.9)
Died	68 (0.0)	68 (0.0)		0 (0.0)	56 (0.0)	85(0.0)		−29 (−51.8)
AMA	3840 (0.9)	1248 (0.8)		2592 (67.5)	2804 (0.7)	2024 (1.0)		780 (27.8)
Chronic/Acute condition								
Acute	38,460 (9.3)	9343 (6.3)	*p* < 0.01	21,117 (75.7)	33,216 (8.8)	14,269 (7.0)	*p* < 0.01	18,947 (57.0)
Chronic	69,943 (15.5)	27,212 (18.4)		36,731 (57.4)	62,662 (16.7)	37,623 (18.5)		25,039 (40.0)
Non-complex chronic condition	62,008 (15.1)	23,162 (15.6)		32,846 (53.0)	54,628 (14.5)	32,233 (15.8)		22,395 (41.0)
Complex chronic condition	7935 (1.9)	4050 (2.7)		3885 (49.0)	8034 (2.1)	5390 (2.6)		2644 (32.9)
NYU ED Algorithm Classification (%)								
Emergent—Not preventable/avoidable	12,510 (3.0)	5022 (3.4)	*p* < 0.01	7488 (59.9)	13,348 (3.6)	7211 (3.5)	*p* < 0.01	6137 (46.0)
Emergent—Preventable/avoidable	15,885 (3.9)	3714 (2.5)		12,171 (76.6)	13,833 (3.7)	4939 (2.4)		8894 (64.3)
Emergent—Primary Care Treatable	81,005 (19.7)	22,514 (15.2)		58,491 (72.2)	75,339 (20.1)	33,643 (16.5)		41,696 (55.3)
Not Emergent	89,726 (21.8)	23,406 (15.8)		66,320 (73.9)	75,498 (20.1)	34,426 (16.9)		41,072 (54.4)
Injuries	93,508 (22.7)	46,780 (31.6)		46,728 (50.0)	90,919 (24.2)	57,817 (28.4)		33,102 (36.4)
Hospital location								
Metro			*p* = 0.40				*p* < 0.01	
Micro/Rural								
Unknown	68,365 (15.9)	24,978 (16.07)		43,387 (63.4)	70,743 (18.1)	35,298 (16.3)		35,445 (50.1)
Hospital Size								
Large	157,683 (36.8)	56,038 (36.1)	*p* < 0.01	101,645 (64.5)	135,552 (34.1)	78,828 (36.3)	*p* < 0.01	56,724 (41.8)
Medium	165,254 (38.6)	61,141 (39.3)		104,113 (63.0)	151,181 (38.6)	83,990 (38.7)		67,191 (44.4)
Small	36,412 (8.7)	13,224 (8.5)		23,188 (63.7)	34,150 (8.2)	18,796 (6.7)		15,334 (44.9)
Unknown	68,365 (15.9)	24,978 (16.1)		43,387 (63.4)	70,743 (18.1)	35,298 (16.3)		35,445 (50.1)
Hospital type								
Children	31,749 (7.4)	13,720 (8.8)	*p* < 0.01	18,029 (56.8)	28,561 (7.3)	19,061 (8.7)	*p* < 0.01	9500 (33.3)
Non-children	327,600 (76.7)	116,683 (75.1)		210,917 (64.3)	292,322 (74.6)	162,553 (75.0)		129,769 (44.4)
Unknown	68,365 (15.9)	24,978 (16.1)		43,387 (63.4)	70,743 (18.1)	35,298 (16.3)		35,445 (50.1)

**Table 2:** Changes in Inpatient Hospital Visits during COVID-19 pandemic by the patient and hospital characteristics.

Variable	April to June				July to September			
	2019 N = 26,156 *n* (%)	2020 N = 17,299 *n* (%)	*p*-Value	Changes (%)	2019 N = 24,475 *n* (%)	2020 N = 20,197 *n* (%)	*p*-Value	Changes (%)
Age								
0–1	1954 (7.8)	1533 (9.3)	*p* < 0.01	421 (21.5)	2160 (9.2)	1634 (8.7)	*p* < 0.01	526 (24.4)
1–5	5464 (21.9)	262 (15.9)		2844 (52.0)	4876 (20.8)	2913 (15.5)		1963 (40.3)
5–10	3896 (15.6)	2113(12.8)		1783 (45.8)	3564 (15.2)	2467 (13.1)		1097 (30.8)
10–14	5081 (20.3)	3388 (20.6)		1693 (33.3)	4464 (21.3)	4010 (21.3)		454 (10.2)
14–17	8610 (34.4)	6802 (41.3)		1808 (21.0)	8340 (41.5)	7806 (41.5)		534 (6.4)
Gender								
Male	12,658 (50.6)	8548 (51.9)	*p* < 0.01	4110 (32.5)	11,693 (50.0)	10,025 (53.2)	*p* < 0.01	1668 (14.3)
Female	12,347 (49.4)	7908 (48.1)		4439 (36.0)	11,711 (50.0)	8805 (46.8)		2906 (24.8)
Race/Ethnicity								
Non-Hispanic White	10,176 (40.7)	7315 (44.5)	*p* < 0.01	2861 (28.1)	9686 (41.4)	8449 (44.9)	*p* < 0.01	1237 (12.8)
Non-Hispanic Black	6136 (24.5)	3809 (23.1)		2327 (37.9)	5862 (25.0)	4283 (22.7)		1579 (26.9)
Hispanic	6996 (28.0)	4028 (24.5)		2968 (42.4)	6178 (26.4)	4643 (24.7)		1535 (24.8)
Others	1697 (6.8)	1304 (7.9)		393 (23.2)	1678 (7.2)	1455 (7.7)		223 (13.3)
Insurance status								
Commercial	7458 (29.8)	5082 (30.9)	*p* < 0.01	2376 (31.9)	6900 (29.5)	5887 (31.3)	*p* < 0.01	1013 (14.7)
Medicaid fee for service	2003 (8.0)	1399 (8.5)		604 (30.2)	2006 (8.6)	1494 (7.9)		512 (25.5)
Medicaid managed care	13,650 (54.6)	8869 (53.9)		4781 (35.0)	12,724 (54.4)	10,215 (54.2)		2509 (19.7)
Others	974 (3.9)	656 (4.0)		318 (32.6)	899 (3.8)	749 (4.0)		150 (19.7)
Uninsured	920 (3.7)	450 (2.7)		470 (51.1)	875 (3.7)	485 (2.6)		390 (16.7)
Physician Referral								
Yes	2439 (9.8)	1774 (10.8)	*p* = 0.02	2439 (9.8)	1774 (10.8)	2439 (9.8)	*p* < 0.01	215 (9.4)
Weekend								
Yes	5459 (21.8)	3634 (22.2)	*p* = 0.15	1825 (34.4)	5194 (22.2)	4026 (21.4)	*p* = 0.19	1168 (22.5)
No	19,546 (78.2)	12,822 (77.9)		6724 (33.4)	18,210 (77.8)	14,804 (78.6)		3406 (18.7)
Discharge disposition								
Routine	23,712 (94.8)	15,447 (93.9)	*p* < 0.01	8265 (34.9)	22,210 (94.9)	17,728 (94.1)	*p* < 0.01	4482 (20.2)
Post-acute	548 (2.2)	461 (2.8)		87 (15.9)	439 (1.9)	480 (2.5)		−41 (9.3)
Home Health	370 (1.5)	242 (0.6)		128 (34.6)	363 (1.6)	300 (1.6)		63 (17.3)
Died	98 (0.4)	101 (0.6)		−3 (−3.1)	101 (0.4)	89 (0.5)		12 (11.9)
AMA	38 (0.2)	26 (0.2)		12 (31.6)	33 (0.1)	37 (0.2)		−4 (12.1)
Chronic/Acute condition								
Acute	12,021 (48.1)	7532 (45.8)	*p* < 0.01	4489 (37.3)	11,596 (49.5)	8375 (44.5)	*p* < 0.01	3221 (27.8)
Chronic	12,188 (48.7)	8593 (52.2)		3595 (29.49)	11,049 (47.2)	10,092 (53.6)		957 (8.7)
Non-complex chronic condition	4954	3352		1602	3956	4018		−62 ()
Complex chronic condition	7234 (28.9)	5241 (31.8)		1993 (27.6)	7093 (30.3)	6074 (32.3)		1019 (14.4)
NYU ED Algorithm Classification (%)								
Emergent—Not preventable/avoidable	1622 (6.5)	978 (5.90	*p* < 0.01	644 (39.7)	1705 (7.3)	1078 (5.7)	*p* < 0.01	627 (36.8)
Emergent—Preventable/avoidable	2057 (8.2)	774 (4.7)		1283 (62.4)	1817 (7.8)	913 (4.8)		904 (49.8)
Emergent—Primary Care Treatable	1109 (4.4)	541 (3.3)		568 (51.2)	1021 (4.4)	734 (3.39)		287 (28.1)
Not Emergent	684 (2.7)	444 (2.7)		240 (35.1)	632 (2.7)	498 (2.6)		134 (21.2)
Injuries	1470 (5.9)	1343 (8.2)		127 (8.6)	1539 (6.6)	1365 (7.2)		174 (11.3)
Hospital location								
Metro	22,019 (84.4)	14,338 (82.9)	*p* < 0.01	7681 (34.9)	20,776 (85.0)	16,718 (82.8)	*p* < 0.01	4058 (19.5)
Micro/Rural	27 (0.1)	29 (0.2)		−2 (7.4)	35 (0.1)	20 (0.1)		15 (42.8)
Unknown	4110 (15.7)	2932 (16.9)		1178 (28.7)	3664 (14.9)	3459 (17.1)		205 (5.6)
Hospital Size								
Large	12,676 (48.5)	8082 (46.7)	*p* < 0.01	4594 (36.2)	12,210 (49.9)	9487 (46.9)	*p* < 0.01	2723 (22.3)
Medium	8217 (31.4)	5342 (30.8)		2875 (35.0)	7399 (30.2)	6151 (30.4)		1248 (16.8)
Small	1153 (4.4)	943 (5.4)		210 (18.2)	1202 (4.9)	1100 (5.4)		102 (8.5)
Unknown	4110 (15.7)	2932 (16.9)		1178 (28.7)	3664 (14.9)	3459 (17.1)		205 (5.6)
Hospital type								
Children	3874 (14.8)	2755 (15.9)	*p* < 0.01		3598 (14.7)	3228 (13.2)	*p* < 0.01	
Non-children	18,172 (69.5)	11,612 (67.1)			17,213 (70.3)	13,510 (55.2)		
Unknown	4110 (15.7)	2932 (16.9)		1178 (28.7)	3664 (14.9)	3459 (17.1)		205 (5.6)

## Data Availability

In this research, limited datasets were used, and datasets are available through the Agency for Health Care Administration, Florida. Website: https://www.floridahealthfinder.gov/researchers/orderdata/order-data.aspx (accessed on 3 April 2021).

## Supplementary Materials

The following are available online at https://www.mdpi.com/article/10.3390/pediatric14010010/s1, Table S1: Descriptive statistics by Medically Underserved Area (MUA) county status, Table S2: Changes in Treat-and-Release Hospital Visits during COVID-19 pandemic by disease systems and common conditions, Table S3: Changes in Inpatient Hospital Visits during COVID-19 pandemic by disease systems and common conditions.
