# Supplementary material for: Impact of the COVID-19 Pandemic on the Pediatric Hospital Visits: Evidence from the State of Florida
Source: Pediatr Rep. 2022 Feb 1;14(1):58–70. doi: 10.3390/pediatric14010010 (PMC8883905; doi:10.3390/pediatric14010010)
Supplement: Supplementary file 1 [file pediatrrep-14-00010-s001.zip › pediatrrep-1528189-supplementary.pdf]

List of Tables and Figures included in the online-only supplement

Table S1: Descriptive statistics by Medically Underserved Area (MUA) county status

Table S2: Changes in Treat-and-Release Hospital Visits during COVID-19 pandemic by disease systems and common conditions

Table S3: Changes in Inpatient Hospital Visits during COVID-19 pandemic by disease systems and common conditions

Table S1. Descriptive statistics by Medically Underserved Area (MUA) county status

Variable	Total N	Full MUA county n, (%)	Partial MUA county, n, (%)	Non-MUA county, n, (%)
County	67	19	12	36
Total Population, N (%)	20598139 (100%)	962842 (4.67)	8982838 (43.60)	10652459 (51.71)
Age Group				
0-17, n (%)	4148552 (20.14)	193454 (20.09)	1896219 (21.10)	2058879 (19.33)
18-65	12385211 (60.13)	565546 (58.73)	5687718 (63.31)	6131947 (57.56)
65+	4064376 (19.73)	203842 (21.17)	1398901 (15.57)	2461633 (23.11)
Race				
White	11041828 (53.60)	547076 (56.81)	3913432 (43.56)	6581320 (61.78)
African American	3218610 (15.62)	108364 (11.26)	1526873 (16.99)	1583373 (14.86)
Hispanic	5134245 (24.92)	232407 (24.13)	2983829 (33.22)	1918009 (18.00)
Others	1203456 (5.84)	74995 (7.78)	558704 (6.12)	569757 (5.35)
Insurance type				
Insured	17543755 (85.17)	785652 (81.59)	7599871 (84.60)	9158232 (85.97)
Not Insured	3064774 (14.88)	187580 (19.48)	1382967 (15.39)	1494227 (14.02)
Social Vulnerability				
Social Vulnerability Index, mean (SD)*	1.00 (1.96)	2.79 (2.29)	0.91(1.44)	1.08 (1.64)
Less than High School	1769489 (8.59)	98032 (10.18)	791510 (8.81)	879947 (8.20)
Household Income Below 100% FPL	2983851 (14.48)	144254 (14.98)	1355783 (15.09)	1483814 (13.93)
Single parent family	653221 (3.17)	28554 (2.96)	310533 (3.45)	314134 (2.94)

SD- Standard Deviation
* Social Vulnerability Index was obtained from CDC SVI Documentation 2018 (https://www.atsdr.cdc.gov/placeandhealth/svi/index.html)
All other data obtained from US American Community Survey 2018

Table S2. Changes in Treat-and-Release Hospital Visits during COVID-19 pandemic by disease systems and common conditions

Variable	April to June			July to September		
	2019 n (%)	2020 n (%)	Changes (%)	2019 n (%)	2020 n (%)	Changes (%)
Body System						
Infectious and parasitic disease	24737 (6.0)	6025 (4.1)	18712 (75.6)	21378 (5.7)	9175 (4.5)	12199 (57.1)
Neoplasms	16 (0.0)	86 (0.1)	80 (48.2)	190 (0.1)	95 (0.0)	95 (50.0)
Metabolic diseases and immunity disorders	2314 (0.6)	849 (0.6)	1465 (63.3)	1930 (0.5)	1052 (0.5)	878 (45.5)
Diseases of blood and blood-forming organs	1029 (0.3)	499 (0.3)	530 (51.5)	1015 (0.3)	603 (0.3)	412 (40.6)
Mental health disorders	3797 (0.9)	2264 (1.5)	1533 (40.4)	3603 (1.0)	3009 (1.5)	594 (16.5)
Nervous system and sense organs disease	37955 (9.2)	9836 (6.6)	28119 (74.1)	31889 (8.5)	13273 (6.5)	18616 (58.4)
Diseases of the circulatory system	1248 (0.3)	576 (0.4)	672 (53.8)	1295 (0.3)	831 (0.4)	464 (35.8)
Diseases of the respiratory system	77597 (18.9)	12344 (8.3)	65253 (84.1)	67635 (0.3)	21669 (10.6)	45966 (68.0)
Diseases of the digestive system	23461 (5.7)	7484 (5.1)	15977 (68.1)	20143 (8.0)	10144 (5.0)	9999 (49.6)
Diseases of the genitourinary System	11446 (2.8)	5549 (3.7)	5897 (51.5)	11298 (5.4)	7382 (3.6)	3916 (34.7)
Complications of pregnancy and the puerperium	1649 (0.4)	1037 (0.7)	612 (37.1)	1740 (0.5)	1290 (0.6)	450 (25.9)
Diseases of the skin and subcutaneous tissue	17960 (4.4)	7294 (4.9)	10666 (59.4)	194490 (5.2)	9630 (4.7)	9860 (50.6)
Diseases of the musculoskeletal system	12626 (3.1)	4541 (3.1)	8085 (64.0)	13157 (3.2)	7134 (3.5)	6023 (45.8)
Congenital anomalies	208 (0.1)	100 (0.1)	108 (51.9)	270 (0.1)	166 (0.1)	104 (38.5)
Conditions originating in the perinatal period	1809 (0.4)	1226 (0.8)	583 (32.2)	2197 (0.6)	1483 (0.7)	714 (32.5)
Symptoms, signs, and abnormal clinical and	77852 (18.9)	26121 (17.6)	51731 (66.4)	65356 (17.4)	37743 (18.5)	27613 (42.3)

laboratory findings, not elsewhere classified						
Injury and poisoning	102983 (25.0)	53013 (35.8)	49970 (48.5)	1000869 (26.9)	65210 (32.0)	35659 (35.4)
Factors influencing health status and contact with health services	7652 (1.9)	6548 (4.4)	1104 (14.4)	7060 (1.9)	10315 (5.1)	-3255 (-46.1)
Disease /condition						
Appendicitis	971 (0.2)	700 (0.5)	271 (63.7)	1005 (0.3)	886 (0.4)	119 (45.3)
Asthma	8151 (2.0)	1475 (1.0)	6676 (27.9)	7712 (2.1)	2475 (1.2)	5237 (11.8)
Seizure	3049 (0.7)	1614 (1.1)	1435 (81.9)	2806 (0.7)	1802 (0.9)	1004 (67.9)
Sickle Cell	520 (0.1)	270 (0.2)	250 (47.1)	497 (0.1)	297 (0.1)	200 (40.2)
Pneumonia	3799 (0.9)	674 (0.5)	3125 (82.3)	2857 (0.8)	722 (0.4)	2135 (74.7)
Cellulitis	3697 (0.9)	1794 (1.1)	1903 (51.1)	4222 (1.0)	2294 (1.1)	1928 (45.7)
Gastroenteritis	12216 (2.9)	4019 (3.0)	8197 (67.1)	11201 (3.0)	6222 (3.1)	4979 (44.45)

Table S3. Changes in Inpatient Hospital Visits during COVID-19 pandemic by disease systems and common conditions

Variable	April to June			July to September		
	2019 n (%)	2020 n (%)	Changes (%)	2019 n (%)	2020 n (%)	Changes (%)
Body System						
Infectious and parasitic disease	1136 (4.5)	570 (3.5)	566 (49.8)	996 (4.3)	692 (3.7)	304 (30.5)
Neoplasms	311 (1.2)	299 (1.8)	12 (3.9)	326 (1.4)	320 (1.7)	6 (1.8)
Metabolic diseases and immunity disorders	933 (3.7)	551 (3.3)	382 (40.9)	777 (3.30	650 (3.5)	127 (16.3)
Diseases of blood and blood-forming organs	855 (3.4)	507 (3.3)	348 (40.7)	847 (3.6)	583 (3.1)	264 (31.2)
Mental health disorders	5190 (20.8)	3600 (21.9)	1590 (30.6)	4303 (18.4)	4324 (23.0)	-21 (-0.5)
Nervous system and sense organs disease	1253 (5.0)	774 (4.7)	479 (38.2)	1159 (5.0)	958 (5.1)	201 (17.3)
Diseases of the circulatory system	271 (1.1)	180 (1.1)	91 (33.6)	275 (1.2)	205 (1.1)	70 (25.5)
Diseases of the respiratory system	3185 (12.7)	708 (4.3)	2477 (77.8)	2806 (12.0)	817 (4.3)	1989 (70.9)
Diseases of the digestive system	1369 (5.5)	845 (5.1)	524 (38.2)	1262 (5.4)	1027 (5.5)	235 (18.6)
Diseases of the genitourinary System	594 (2.4)	413 (2.5)	181 (30.5)	628 (2.7)	451 (2.4)	177 (28.2)
Complications of pregnancy and the puerperium	511 (2.0)	470 (2.9)	41 (8.0)	620 (2.6)	556 (3.0)	64 (10.3)
Diseases of the skin and subcutaneous tissue	623 (2.5)	343 (2.1)	280 (44.9)	644 (644)	417 (2.2)	227 (35.2)
Diseases of the musculoskeletal system	784 (3.1)	536 (3.1)	248 (31.6)	731 (3.1)	649 (3.4)	82 (11.2)
Congenital anomalies	659 (2.6)	500 (3.0)	159 (24.1)	703 (3.0)	583 (3.1)	120 (17.1)
Conditions originating in the perinatal period	1518 (6.1)	1179 (7.2)	339 (22.3)	1688 (7.2)	1290 (6.9)	398 (23.6)
Symptoms, signs, and abnormal clinical and laboratory findings, not elsewhere classified	701 (2.8)	407 (2.5)	294 (41.9)	691 (3.0)	472 (2.5)	219 (31.7)

Injury and poisoning	1942<br>(7.8)	1814<br>(11.0)	128<br>(6.6)	1964<br>(8.4)	1864<br>(9.9)	100<br>(5.1)
Factors influencing health status and contact with health services	734<br>(2.9)	659<br>(4.0)	75<br>(10.2)	697<br>(3.0)	678<br>(3.6)	19<br>(2.7)
Disease /condition						
Appendicitis	429<br>(1.7)	464<br>(2.8)	-35<br>(-8.2)	450<br>(1.9)	424<br>(2.3)	26<br>(5.8)
Asthma	47<br>(0.2)	33<br>(0.2)	14<br>(29.8)	46<br>(0.2)	41<br>(0.2)	5<br>(10.9)
Seizure	473<br>(1.9)	272<br>(1.7)	201<br>(42.5)	438<br>(1.9)	351<br>(1.9)	87<br>(19.9)
Sickle Cell	455<br>(1.8)	260<br>(1.6)	195<br>(42.9)	437<br>(1.9)	288<br>(1.5)	149<br>(34.1)
Pneumonia	450<br>(1.8)	105<br>(0.6)	345<br>(76.7)	310<br>(1.3)	82<br>(0.4)	228<br>(73.5)
Cellulitis	346<br>(1.3)	172<br>(1.0)	174<br>(50.2)	358<br>(1.5)	215<br>(1.1)	143<br>(39.9)
Gastroenteritis	1051<br>(3.4)	634<br>(3.3)	417<br>(39.7)	989<br>(3.1)	810<br>(3.2)	170<br>(18.1)
